# The potential of posterior cruciate ligament buckling phenomenon as a sign for partial anterior cruciate ligament tears

**DOI:** 10.1007/s00402-024-05270-0

**Published:** 2024-03-16

**Authors:** Mehmet Ali Tokgoz, Ethem Burak Oklaz, Oguzhan Ak, Elif Banu Guler Oklaz, Muhammet Baybars Ataoglu, Ulunay Kanatli

**Affiliations:** 1 Department of Orthopaedics and Traumatology, Gazi University Faculty of Medicine, Emniyet Mahallesi, Mevlana Bulvarı No: 29 Yenimahalle, Ankara, Turkey; 2 Department of Radiology, Bilkent City Hospital, Ankara, Turkey

**Keywords:** Partial anterior cruciate ligament tears, Posterior cruciate ligament buckling phenomenon, Magnetic resonance imaging, Knee arthroscopy

## Abstract

**Introduction:**

Diagnosis of a partial tear of the anterior cruciate ligament (ACL) can be challenging with physical examination and imaging techniques. Although magnetic resonance imaging (MRI) has high sensitivity and specificity for diagnosing complete ACL tears, its effectiveness may be limited when it is used to diagnose for partial tears. The hypothesis of the present study is that the posterior cruciate ligament (PCL) buckling phenomenon, which is a secondary sign of complete ACL tears on MRI, may be a useful method for diagnosing partial ACL tears.

**Materials and methods:**

The data of 239 patients who underwent knee arthroscopy in a single institution between 2016 and 2022 were analyzed retrospectively. Patients were divided into three groups based on the condition of their ligaments: partial tears, complete tears and intact ligaments. To evaluate the buckling phenomenon on sagittal T2-weighted MRI, measurements of the posterior cruciate ligament angle (PCLA) and the posterior cruciate ligament-posterior cortex angle (PCL-PCA) were conducted in each group. Subsequently, the ability of these two measurement methods to distinguish partial tears from the other groups was assessed.

**Results:**

Both methods provided significantly different results in all three groups. Partial tears could be distinguished from intact ligaments with 86.8% sensitivity, 89.9% specificity when PCLA < 123.13° and 94.5% sensitivity, 93.2% specificity when PCL-PCA < 23.77°. Partial tears could be distinguished from complete tears with 79.5% sensitivity, 78.4% specificity when PCLA > 113.88° and with 86.1% sensitivity, 85.3% specificity when PCL-PCA > 16.39°.

**Conclusion:**

The main finding of the present study is that the PCLA and PCL-PCA methods are useful on MRI for diagnosing partial ACL tears. PCLA value between 113°-123° and PCL-PCA value between 16°-24° could indicate a partial ACL tear. With these methods, it is possible to distinguish partial tears from healthy knees and reduce missed diagnoses. In addition, the differentiation of partial and complete tears by these methods may prevent unnecessary surgical interventions.

**Level of evidence:**

Level III.

**Supplementary Information:**

The online version contains supplementary material available at 10.1007/s00402-024-05270-0.

## Introduction

Partial anterior cruciate ligament (ACL) injury constitutes 10–27% of isolated ACL injuries [1]. There are different views on the definition of this injury. Some authors define the injury based on damage to the anteromedial (AM) and posterolateral (PL) bundles of the ACL, identified by their tibial insertion sites, while others define it based on the percentage of ACL fibers that are torn [1–5]. Despite the controversy over its definition, tears in which the ligament is not completely damaged are generally considered partial tears [3]. In clinical practice, physical examination and imaging techniques such as magnetic resonance imaging (MRI) are commonly used for the diagnosis of this condition. As the Lachman test and pivot-shift test have limited accuracy in detecting partial tears, the value of imaging in these patients is even greater [1, 6]. MRI has a sensitivity of 92–100% and a specificity of 85–100% for diagnosing complete tears, but these rates are lower for incomplete tears [7, 8]. Instrumental laxity measurements may help to differentiate partial tears from complete tears, but are not useful in differentiating partial tears from intact ACLs unless there is a severe damage [9, 10]. For this reasons, these injuries are usually diagnosed by arthroscopic examination.

Differentiating partial tears is crucial for the patient’s prognosis. Unnecessary surgical treatments could be prevented by distinguishing patients with partial tears who have the potential for recovery by conservative treatment from those with complete tears [10–13]. Moreover, distinguishing between patients with partial tears and those with intact ACLs can help avoid missed diagnoses and guide appropriate conservative or surgical treatment [10, 14]. The differentiation between partial and complete tears is also very important when deciding on the surgical technique. While ACL reconstruction is often the main surgical modality for complete tears, there are also options for treating partial tears, such as AM or PL bundle reconstruction [15, 16] and primary repair [17–19]. The distinction between partial and complete tears is therefore crucial in preoperative planning. To this end, several studies have been published using 3T MRI and specific MRI findings to achieve a more accurate diagnosis [8, 20, 21]. Although these studies have been helpful in identifying partial tears, there is currently a need for research that demonstrates high sensitivity and specificity in diagnosing them.

**Fig. 1:**
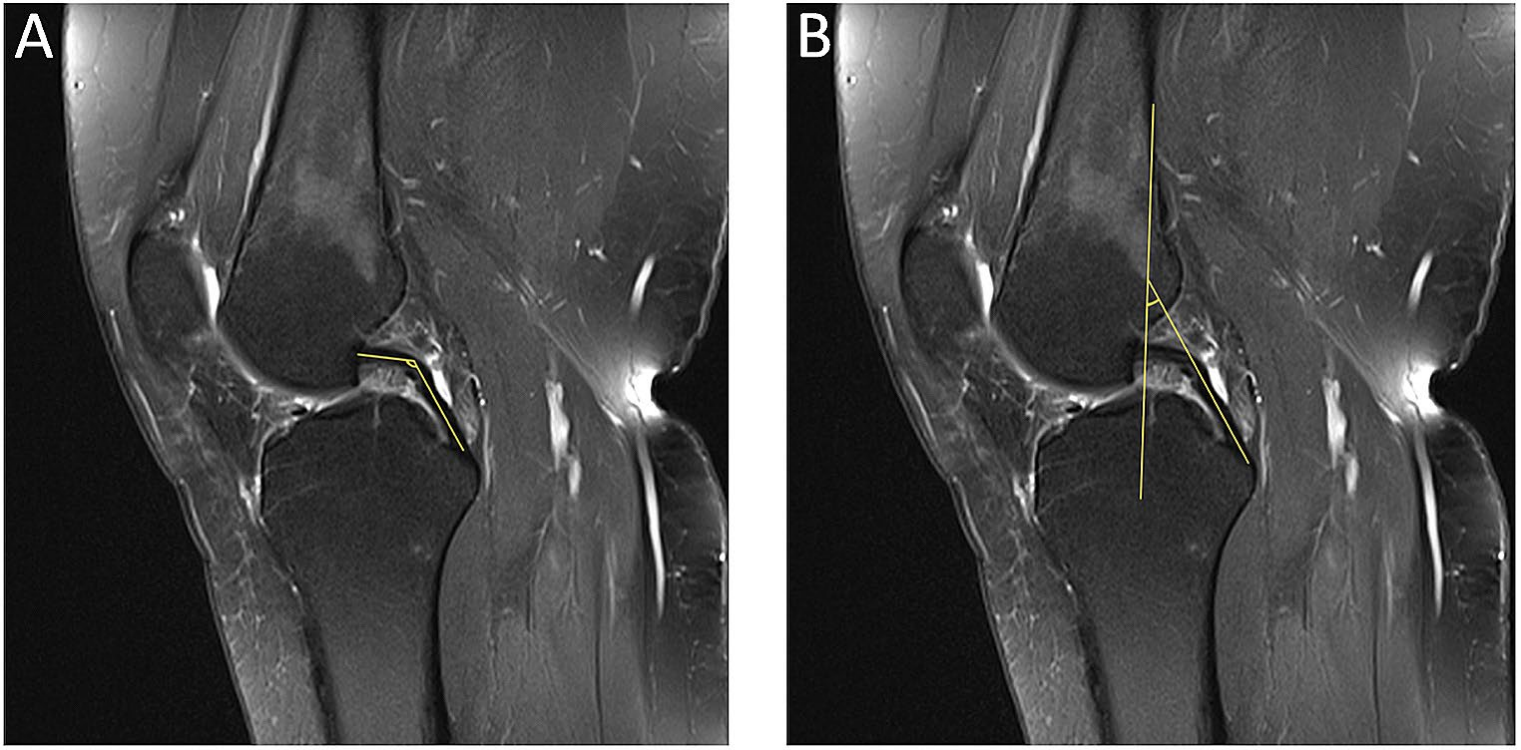
Sagittal T2-weighted MRI of the knee showing PCLA **(a)** and PCL-PCA **(b)** views

A review of the literature on secondary MRI findings revealed that, particularly in recent years, new studies have been conducted on the posterior cruciate ligament (PCL) buckling phenomenon [22, 23]. In sagittal MRI evaluations of patients with ACL injuries, the vertical component of the PCL becomes more upright as a result of increased anterior tibial translation (ATT). The PCL angle (PCLA) is the most commonly utilized method for assessing the degree of this finding (Fig. 1a) [24]. A more recent study also described the PCL-posterior cortex angle (PCL-PCA), which has been shown to have higher sensitivity and specificity in determining the degree of this finding (Fig. 1b) [23].

The purpose of this study is to assess the applicability of these two methods for diagnosing partial ACL tears, which are difficult to detect by MRI. We hypothesize is that these methods may be useful in the differential diagnosis of partial tears.

## Methods

The study protocol was approved by the University Ethics Committee (Decision E-77082166-604.01.02-673921, research code: 2023 − 649) and adhered to the guidelines of the World Medical Association Declaration of Helsinki. In this study, video recordings and MRIs of patients who underwent arthroscopic surgery at a single institution between 2016 and 2022 were evaluated retrospectively. The data of 285 patients were collected prospectively. Patients with inflammatory arthritis, PCL or collateral ligament injury, patellofemoral malalignment, a history of previous knee surgery, bucket handle meniscus tears, meniscus root tears and complex meniscus tears were excluded from the study. The remaining 239 patients were included in the study. Demographic data, including age,sex and body mass index (BMI) were analyzed.

In the arthroscopic examination, both ACL bundles were examined using a probe. For a more accurate assessment, the PL bundle was also evaluated at Cabot’s position (figure of four position) [25]. According to this, ACLs were categorized into 3 groups: group 1 A (46 patients) was considered to have an injury to the AM bundle with an intact PL bundle and group 1B (28 patients) was considered to have an injury to the PL bundle with an intact AM bundle, group 2 (74 patients) was considered to have complete tears and group 3 (91 patients) was considered to have intact ACLs.

MRI was performed on a 1.5-Tesla system (Signa, HiSpeed; General Electric Medical Systems, Milwaukee, WI, USA) with the patient in a supine position and the knee in extension. The imaging protocol included sagittal T2-weighted (time to response [TR]/time to echo [TE]: 2460/41) images. The field of view was 25 cm and the slice thickness/interslice gap was 3e4/0–1 mm in all sequences. In a small number of patients (6.7%, *n* = 16) in whom the PCL could not be completely evaluated in a single sagittal slice, we used thin-slice 3D fast spin echo T2-weighted images, which are available in the routine knee MR protocol in our clinic and allow multiplanar reconstruction and angle correction.

The PCLA was determined to be the angle between a line through the central portion of the femoral insertion of the PCL and a line through the central portion of the tibial insertion of the PCL (Fig. 1a) [24]. The PCL-PCA method was evaluated on the sagittal plane view at the most lateral portion of the tibial insertion of the PCL. The angle was measured between a line drawn from the posterior diaphyseal cortex of the femur and a line parallel to the central portion of the most vertical part of the PCL (Fig. 1b) [23, 26]. Mean PCLA and PCL-PCA compared among the groups. Subsequently, the sensitivity and specificity of these two methods for distinguishing partial tears from those of the other groups were assessed. The medial posterior tibial slope (MPTS) and lateral posterior tibial slope (LPTS) were measured from sagittal MR images of the patients [27]. The relationships of these tibial slope values with the PCLA and PCL-PCA were evaluated.

MRIs were evaluated by a radiologist and a knee surgeon who were blinded to the former radiologic reports and arthroscopic results. Arthroscopic surgery video recordings were evaluated by two knee surgeons with at least 10 years of experience. To investigate intraobserver reliability, the same observer reevaluated all MRI or video records at intervals of more than 2 weeks from the initial evaluation. To evaluate interobserver reliability, another observer similarly evaluated all the video records or MRIs randomly.

The sample size calculation was performed with an aim to account for a 20% difference in complication rates between the two groups. The significance level (alpha) was set at 5% and the desired statistical power was 80%. Consequently, each group initially included 28 participants. However, in order to enhance the statistical power of the study, a decision was made to screen all eligible patients from the archive of the current institute where the study was conducted [23].

### Statistical analysis

Statistical analysis and graphics editing were performed using IBM SPSS 26.0 (IBM Corp., Armonk, NY., USA). Measurement data were reported as mean ± standard deviation (range of values), while nominal data were presented as numbers and percentages. To attain both inter-rater and intra-rater reliability, a mixed-effects model was employed, treating reviewer-rater discrepancies as fixed effects. The assessment utilized the inter-class correlation coefficient (ICC) for absolute agreement, taking into account the mean of the scores provided by the two reviewers. Consistently high inter-rater agreement was observed for all measures, as indicated by ICC values exceeding 0.8. The MPTS and LPTS data were split based on the intactness of the anterior cruciate ligament. Subsequent analysis, employing Spearman’s rho correlation coefficient, sought to determine the relationships PCLA and PCL-PCA with both medial and lateral posterior tibial slope measurements.

The chi-square test was employed to compare categorical variables, the ANOVA test was used for comparing binary measurement data in independent groups, and the Kruskal-Wallis test was utilized when normal distribution assumptions were not met. Post-hoc pairwise analyses were conducted following multiple group comparisons. A significance level of *p* < 0.05 was considered statistically significant, and Bonferroni correction was applied to adjust p-values for the pairwise analyses. ROC analysis was employed to determine the cut-off measurements complete and partial tears. The performance of the measurements was assessed using the area under the ROC curve (AUC). AUC values between 0.9 and 1 were classified as excellent, values between 0.8 and 0.9 as good, values between 0.7 and 0.8 as fair, values between 0.6 and 0.7 as poor, and values between 0.5 and 0.6 as failed [28, 29].

## Results

A retrospective review was conducted on a total of 239 patients who were included in the study. Of these patients, 171 were male and 68 were female, with a mean age of 33.09 ± 11.70 years and a median age of 32.5 years (range, 13–52) at the time of surgery. There was no statistically significant difference between the groups in terms of mean age or BMI (*p* = 0.3 and *p* = 0.2, respectively) (Table 1). There were 67 meniscal injuries, 12 patellofemoral disorders and 12 osteochondral defects in the intact ACL group.

**Table 1:** Comparison of measurement methods and demographics in 3 groups

	Group 1		Group 2	Group 3	
	A	B			
n	46	28	74	91	
Age	34.33 ± 14.65	31.05 ± 13.34	30.8 ± 8.83	33.05 ± 10.91	
Gender (F/M)	15/31	8/20	7/67	38/53	
BMI	24.1 ± 2.4	23.7 ± 2.6	24.3 ± 2.2	24.6 ± 2.1	
PCLA	116.47 ± 4.89	118.35 ± 5.47	109.97 ± 5.90	129.11 ± 5.85	
PCL-PCA	19.73 ± 3.30	18.75 ± 3.15	14.26 ± 2.47	31.25 ± 5.24	
Medial PTS	6.30 ± 2.50	6.39 ± 1.96	6.52 ± 2.15	6.11 ± 2.32	
Lateral PTS	6.71 ± 2.43	6.87 ± 1.98	6.97 ± 2.12	5.61 ± 2.22	

BMI: Body Mass Index, PCLA: Posterior Cruciate Ligament Angle, PCL-PCA: Posterior Cruciate Ligament-Posterior Cortex Angle, PTS: Posterior Tibial Slope

The mean values of the PCLA and PCL-PCA were found to be significantly different among the three groups of patients (*p* < 0.05) (Table 1). There was no significant difference in the mean values of PCLA and PCL-PCA between group 1A and group 1B (*p* = 0.24) (Table 1). The analysis of MPTS and LPTS results by group revealed no statistically significant correlation between PCLA and PCL-PCA with both medial or lateral PTS (*p* > 0.05 for all values) (Table 1).

**Fig. 2:**
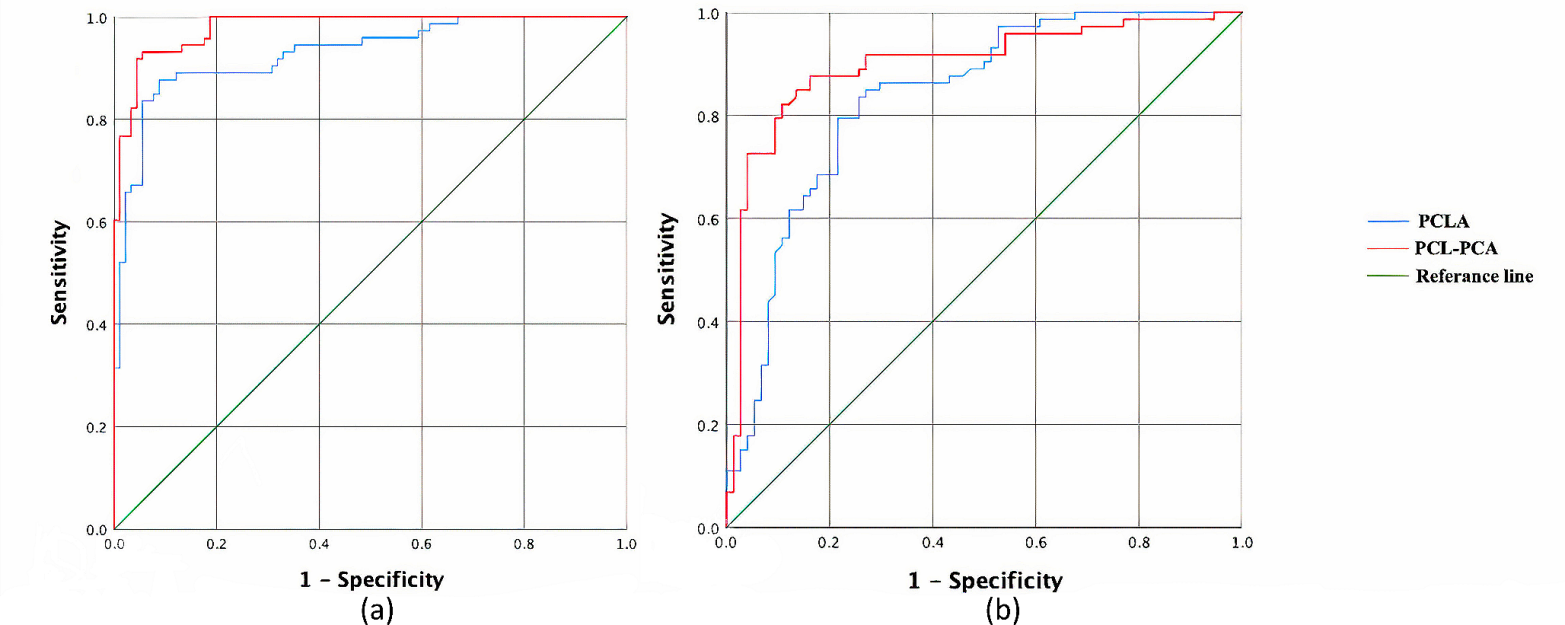
ROC curves to compare the ability of the PCLA and PCL–PCA methods to discriminate between group 1- group 3 **(a)** and group 1- group 2 **(b)**

When evaluating knees with partial tears and intact ligaments, both the PCLA and PCL-PCA methods were found to differentiate partial tears with high sensitivity and specificity by determining specific cut-off values (PCLA: < 123.13°, PCL-PCA: < 23.77°). Similarly, the same methods could be used to differentiate partial tears from complete tears (Cut-off values; PCLA: > 113.88°, PCL-PCA: > 16.39°). The PCL-PCA angle is slightly more effective at making this distinction (Fig. 2; Table 2).

**Table 2:** Comparison of the PCLA and PCL–PCA methods in their ability to discriminate between groups

	AUC (95% CI)	p	Cut-off	Sensitivity (%)	Specificity (%)	+LHR	PPV (%)	NPV (%)	SEM	ES
Partial ACL Tear - Intact ACL										
PCLA	0.930 (0.890–0.970)	< 0.001	123.13°	86.8	89.9	7.92	86.8	89.0	0.020	0.93
PCL-PCA	0.980 (0.963–0.996)	< 0.001	23.77°	94.5	93.2	13.64	94.5	93.2	0.008	0.98
Partial ACL Tear - Complete ACL Tear										
PCLA	0.835 (0.770–0.901)	< 0.001	113.88°	79.5	78.4	3.67	79.5	78.4	0.033	0.83
PCL-PCA	0.899 (0.844–0.953)	< 0.001	16.39°	86.1	85.3	6.28	84.9	86.5	0.028	0.89

ACL: Anterior Cruciate Ligament, PCLA: Posterior Cruciate Ligament Angle, PCL-PCA: Posterior Cruciate Ligament-Posterior Cortex Angle, +LHR: Positive Likelihood Ratio, PPV: Positive Predictive Value, NPV: Negative Predictive Value, SE: Standart Error of Measurement, ES: Effect Size

## Discussion

The most important finding of this study is that the PCL buckling phenomenon, which is used to diagnose complete ACL tears, can also be a useful method for differentiating partial tears. PCLA between 113°- 123° and PCL-PCA between 16°- 24° could be a sign for a partial ACL tears. The differentiation between partial tears with an intact ACLs and complete tears is crucial for determining patient prognosis and treatment methods. Conservative treatment can be effective for partial tears in children, adolescents and adults without high activity levels, especially for those with an intact synovial sheath and without symptomatic knee instability [10–12]. In these patients, distinguishing between a partial tear and a complete tear may be crucial in avoiding unnecessary surgery. On the other hand, conservative treatment may give less satisfactory results for high-demand young patients who are performing contact sports and want to return to sports in the short term. In these patients, if a partial tear is diagnosed early, repair may be performed without progression to a complete tear, and better clinical results can be achieved [14, 30–32]. As physical examination findings may not be clear in patients with partial tears, diagnostic imaging is usually necessary to diagnose and determine treatment decisions.

Despite the need for imaging in diagnosis, studies in the literature suggest that MRI, which is the gold standard method for determining ACL injuries, is inadequate for diagnosing partial tears. Van Dyck et al. investigated whether the findings used for the diagnosis of complete tears by MRI could also be applied to patients with partial tears [20]. This study revealed that abnormal ligament signal intensity can contribute to diagnosis with a sensitivity of 71%, but findings such as discontinuity and abnormal morphology have low sensitivity in assessing partial tears. In addition, the same study indicated that secondary MR signs, which are valuable in diagnosing ACL injuries, are not sufficient for differentiating between partial and complete tears. Lefevre et al. suggested that the appearance of a mobile stump resulting from a partial tear on MR imaging could be useful in the diagnosis [21]. Despite the high specificity of this finding, its sensitivity was found to be quite low. Günaydin et al. reported that partial tears were more easily diagnosed on MR images taken while the patient was in the prone position and with the knee flexed [33]. Although this method provides better images for assesing the ACL, the challenge of standardization may make its general use difficult. Other studies on this topic have also noted the difficulty of diagnosing partial ACL tears by using MRI and have stated that there is a need for new perspectives and research on this subject [1, 9].

We suggest that the utilization of the PCL buckling phenomenon holds promise as a valuable method for assessment. PCLA is commonly used to evaluate this phenomenon. Different cut-off values of this angle were previously defined (< 105–118°) and found to be useful for diagnosis with a sensitivity of 52–74% and a specificity of 71–94% [23, 24, 34, 35]. In our study, similar to the values in the literature, the probability of diagnosing a complete tear increased significantly when the PCLA decreased below 113°. In the differential diagnosis of partial tears, which is our main objective, this method was able to detect tears significantly between 113–123°.

Siboni et al. described the PCL-PCA method in a study evaluating PCL buckling phenomenon in 24 patients with ACL injury [23]. In this study, when ACL injuries were not categorized as partial or complete, the PCL-PCA cut-off value was found to be 22.65°, and it has been reported that ACL injuries can be diagnosed with 71% sensitivity and 88% specificity in knees with values less than this cut-off. An important result of this study was that the PCL-PCA method gave better results than did the PCLA method in evaluating the buckling phenomenon. The main difference between our study and this one is that we categorized ACL tears as partial and complete, and we included a larger number of patients. Our results support the findings of Siboni et al. because the PCL-PCA method has greater sensitivity and specificity than PCLA method for differentiating partial tears from complete tears and from intact ACLs.

The present study revealed no relationship between PCLA or PCL-PCA values and tibial slope. This may be because the tibial slope was correlated with preoperative anterior knee laxity, whereas the buckling phenomenon was not correlated with the degree of laxity [36–40]. Since there is limited information on this subject in the literature, more studies are needed to evaluate the PCL buckling phenomenon in relation to femur and tibia morphology.

Zantop et al. demonstrated that there was a significant increase in ATT at 60° and 90° of flexion in patients who underwent AM bundle transection, and this increase was significant at 30° of flexion in patients who underwent PL bundle transection [41]. On the basis of our study, we believe that the reason for the nonsignificant difference in the mean PCLA and PCL-PCA between patients with AM and PL bundle tears is that the MRI were taken during knee extension.

One of the main advantages of these methods is that they are easy to assess on MRI. Unlike secondary signs of ACL tears, which can be challenging to identify on MRI, PCL buckling is readily visible in the sagittal view. This clear visualization of PCL buckling on MRI scans can be highly beneficial for diagnosing partial tears.

There are several limitations of our study. This is a retrospective study in which the data were collected prospectively. The results of the Lachman test, pivot shift test and laxity measurements of the patients under anesthesia are not available in our study. Therefore, no correlation analysis could be performed between these clinical instability tests and PCLA or PCL-PCA values. However, Chang et al. did not find any relationship between PCL buckling with Lachman test and pivot shift test in their study [42]. The PCL buckling phenomenon becomes more noticeable with chronic ACL injury [23]. Unfortunately, we did not have enough information about the date of the patients’ injuries in our study. Therefore, we do not have information on the time between the injury and the dates of the MRI examination and surgery in the conducted study. Another limitation of presented study is that we did not investigate the relationship between the morphological structure of the tibia and femur with the PCLA or PCL-PCA methods, except for the PTS. Further studies are needed to investigate the relationship between measurement methods that examine the PCL buckling phenomenon and knee ligaments and bone morphology.

## Conclusion

Evaluation of the PCL buckling phenomenon using the PCLA and PCL-PCA methods may be useful in the differential diagnosis of partial tears. PCLA between 113° − 123° and PCL-PCA between 16° − 24° could indicate a partial ACL tear. With these methods, it is possible to distinguish partial tears from healthy knees and reduce missed diagnoses. In addition, the differentiation of partial and complete tears by these methods may prevent unnecessary surgical interventions.

### Electronic supplementary material

Below is the link to the electronic supplementary material.

Supplementary Material 1
